# Role of inflammation in oral carcinogenesis (Part II): CD8, FOXP3, TNF-α, TGF-β and NF-κB expression

**DOI:** 10.3892/ol.2013.1302

**Published:** 2013-04-11

**Authors:** MARTA RABELLO PIVA, LÉLIA BATISTA DE SOUZA, PAULO RICARDO SAQUETE MARTINS-FILHO, CASSIANO FRANCISCO WEEGE NONAKA, THIAGO DE SANTANA SANTOS, EMANUEL SÁVIO DE SOUZA ANDRADE, DIOGO PIVA

**Affiliations:** 1 Department of Oral Pathology, Federal University of Sergipe, Sanatório, Aracaju 49060-100, Sergipe;; 2 Department of Oral Pathology, Federal University of Rio Grande do Norte, Natal 59072-970, Rio Grande do Norte;; 3 Department of Health Education, Federal University of Sergipe, Lagarto 49400-000, Sergipe;; 4 Department of Oral and Maxillofacial Surgery and Periodontology, Ribeirão Preto Dental School, University of São Paulo, Ribeirão Preto 14040-904, São Paulo;; 5 Department of Oral and Maxillofacial Surgery, Pernambuco University, Camaragibe 54753-220, Pernambuco;; 6 Department of Surgery, Federal University of São Paulo (EPM/UNIFESP), Vila Clementino, São Paulo 04023-062, Brazil

**Keywords:** carcinoma, cytokines, inflammation, immunohistochemistry

## Abstract

Due to the frequent presence of inflammation in cases of carcinoma and its use as a parameter for the assessment of tumor aggressiveness, the role of inflammation in oral carcinogenesis was investigated. This was performed by evaluating the expression of cellular markers, cytokines and nuclear transcription factors that identify the cells that participate in the antitumor defense in cases of oral epithelial dysplasia (OED) and oral squamous cell carcinoma (OSCC). A semi-quantitative immunohistochemical analysis was performed for the transcription factors cluster of differentiation 8 (CD8), forkhead box P3 (FOXP3), transforming growth factor (TGF)-β, tumor necrosis factor (TNF)-α and nuclear factor κ-light chain enhancer of activated B-cells (NF-κB), in cases of OED and OSCC. CD8, TGF-β, TNF-α and NF-κB participated in the processes of tumor transformation and progression. The presence of inflammatory infiltrate in cases of OED favors the transformation and invasion process when stromal TNF-α and NF-kB are overexpressed, as NF-kB activated by TNF-α during inflammation predisposes the lesion to transformation, functioning as a link between inflammation and cancer. The control of these inflammatory mediators may prevent malignant transformation in the oral cavity.

## Introduction

Cancer arises from the uncontrolled dissemination and spread of clones of transformed cells, which should be recognized by the immune system before transforming into a tumor. Although it has been demonstrated that the immune system reacts to many tumors and at least slows down the progression, it is not yet known how immune reactions are used to destroy tumors in a specific manner. Moreover, one must take into account the capacity of tumor cells to evade or overcome the defense mechanisms of the host (1). Remodeling of the extracellular matrix and basal membrane confined to the pericellular microenvironment may be the first step toward invasion (2). Escape from the action of the immune system results in the rapid progression of cancer, requiring immunotherapy to potentiate the antitumor response of the host and avoid dissemination (3).

The effector mechanisms of the immune response begin with the bonding of a signaling agent to a specific receptor on the cell surface, which sends signals to the nucleus through signal transduction pathways, where regulating factors known as transcription factors promote specific alterations in the regulation of gene expression.

Nuclear factor κ-light chain enhancer of activated B-cells (NF-κB) is a transcription factor activated in response to signals from the T-cell receptor (TCR) and is essential in the synthesis of cytokines. In the resting T-cell, this protein is found in the cytoplasm associated with inhibitor proteins (IκBs). Signals from the TCR induce phosphorylation via IκB kinases, which is followed by the insertion of multiple copies of a small protein called ubiquitin, which releases NF-κB. This enters the nucleus where it contributes to the transcriptional activation of several genes of cytokines and cytokine receptors. NF-κB is involved in the activation of T-cells, contributing to the transcription of interleukin 2 (IL-2) and the response of many cell types to pro-inflammatory cytokines, such as tumor necrosis factor (TNF), IL-1 and bacterial lipoproteins (4).

The maintenance of activated NF-κB during inflammation predisposes a tumor to malignant transformation. NF-κB could be used to inhibit tumor transformation, but for such it would be necessary to interfere in its physiological role in both immunity and/or inflammation and homeostasis (5).

Human tumors activate cluster of differentiation 4 (CD4) or CD8 lymphocytes, depending on the processing pathway for triggering the immune response. Control of the tumor depends both on the magnitude of the initial immune response and the capacity to sustain this response for a prolonged period of time (6). The main antitumor defense mechanism is the death of tumor cells by CD8 T-lymphocytes, also known as cytotoxic T-lymphocytes. They have the ability to recognize and kill potentially malignant cells that express peptides derived from mutant cell proteins or oncogenic viral proteins associated with major histocompatability complex (MHC) class I.

The Treg cell line is a T-lymphocyte subtype that has the role of inducing and maintaining immunological tolerance and the finalization of the immune response. A deficiency or reduction in this cell type leads to an auto-immune disease (7,8). However, a group of adaptable Treg cells (Th3) become mature in peripheral tissues under antigen stimulation and/or co-stimulation, exercising a suppressive function through the secretion of IL-10 and transforming growth factor-β (TGF-β).

Forkhead box P3 (FOXP3) is a protein responsible for the regulation of the function and development of Treg cells and has been used in their detection (9–11). According to Hori *et al*(7), FOXP3 is the best marker for Treg cells.

In normal tissue, TGF-β regulates cell growth and differentiation. The autocrine reduction of TGF-β in keratinocytes has been found to lead to papillomatous lesions that transform into carcinomas (12). The cancer cells develop partial or complete resistance to this inhibition, however (13). TGF-β often functions as a tumor suppressor in the early stages of carcinogenesis and later becomes a promoter in the progression of the tumor and metastasis (14,15). The negative regulation or damage to the disposition of the receptors on the surface of cytolytic T-lymphocytes allows tumor cells to escape the inhibitory effects of TGF-β (16).

This study assessed the role of inflammation in oral carcinogenesis through the investigation of cellular markers, cytokines and nuclear transcription factors that identify the cells that participate in antitumor defense in cases of oral epithelial dysplasia (OED) and oral squamous cell carcinoma (OSCC).

## Materials and methods

### Selection criteria

A total of 20 cases of OED and 40 cases of OSCC were randomly selected from the archives of the Pathological Anatomy Service of the Federal University of Sergipe and the Pathological Anatomy Laboratory of the Oral Pathology Sector of the Federal University of Rio Grande do Norte in Brazil. The study received approval from the Research Ethics Committee of the latter institution (process no. 233/2007).

### Histology

The hematoxylin and eosin-stained slides were viewed under a light microscope and examined in a double-blind manner by two histopathologists. The criteria of the World Health Organization (WHO) were used for the histological grading of the dysplasia (17). Carcinomas were classified as stage I (low-grade) or stage II (high-grade) according to the method proposed by Piva *et al*(18).

### Immunohistochemistry

Immunohistochemical analysis was performed for CD8, FOXP3, TGF-β, TNF-α and NF-κB in cases of dysplasia and carcinoma. Paraffin-embedded specimens were cut (3 *μ*m) and the slices were mounted on glass slides prepared with an organosilane-based adhesive (3-amino-propyltriethoxysilane, Sigma Chemical Co., St. Louis, MO, USA). Antibodies directed against the proteins studied were applied to the histological slices, according to the specifications displayed in Table I. For negative controls, the primary antibodies were omitted. For positive controls, previously tested breast cancer and periapical granuloma specimens were used. Following the classification of the cases as dysplasia and carcinoma, a semi-quantitative analysis was performed on the markers, cytokines and nuclear transcription factor expression, with reactions of a brown coloration considered positive, regardless of intensity. The percentage of positive cells was calculated for CD8 and FOXP3 for stromal cells, NF-κB for epithelial cells and TGF-β and TNF-α for both epithelial and stromal cells. Labeled cells were classified as low expression with <5%, as moderate expression with 5–50% and as high expression with >50%, following the criteria proposed by Abbas *et al*(19).

### Statistical analysis

The statistical analysis was performed using SPSS 13.0 for Windows (SPSS Inc., Chicago, IL, USA). The Mann-Whitney U test was used to determine the hypothesis of equality in the expression of the markers, cytokines and nuclear transcription factors in relation to the type of lesion and histological grade of carcinoma. The Kruskal-Wallis test was used to compare the expression of CD8, FOXP3, TGF-β, TNF-α and NF-κB between the grades of dysplasia. The Spearman’s rank correlation coefficient was used to investigate the correlation between inflammatory infiltrate intensity and positivity of CD8, FOXP3, TGF-β, TNF-α and NF-κB. P<0.05 was considered to indicate a statistically significant result.

## Results

### Histology and immunohistochemistry

**Figure 1:**
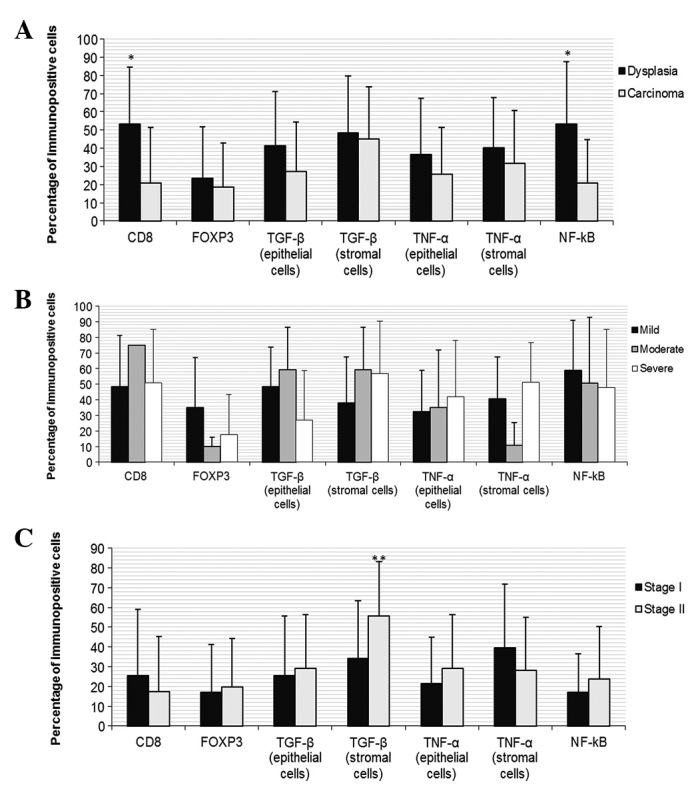
Expression of CD8, forkhead box P3 (FOXP3), tumor growth factor-β (TGF-β), tumor necrosis factor-α (TNF-α) and nuclear factor κ-light chain enhancer of activated B-cells (NF-κB) according to the type of lesion (A), histologic grades of dysplasia (B) and stages of oral squamous cell carcinoma (C). Percentage of immunopositive cells are shown in the hatched columns. Bars represent the standard error of the mean. The expression of CD8 and NF-κB in dysplasia was found to be higher than in carcinoma (^*^P<0.0001). The expression of stromal TGF-β in stage II carcinoma was found to be higher than in stage I (^**^P= 0.022).

CD8 and NF-κB expression were found to be significantly higher in the dysplasia group (Fig. 1A). However, no differences were noted in the expression of CD8, FOXP3, TGF-β, TNF-α and NF-κB between degrees of dysplasia (Fig. 1B). The immunohistochemical expression of cellular markers, cytokines and nuclear transcription factor was no different between stages of carcinoma, with the exception of the stromal TGF-β, which was higher in stage II lesions (Fig. 1C).

TNF-α in both epithelial and stromal cells and NF-κB exhibited a direct, statistically significant correlation with the intensity of the inflammatory infiltrate in the cases of dysplasia. In the cases of carcinoma, the intensity of the inflammatory infiltrate was directly and significantly correlated with CD8 and stromal TNF-α expression. In contrast, an inverse correlation between the inflammatory infiltrate and the expression of epithelial TNF-α was observed in carcinomas (Table II).

## Discussion

Comparative studies have demonstrated that significant molecular alterations occur in the progression of OED to invasive carcinoma, but not between histological grades of OSCC (19). The present study corroborates these findings, since the expression of cytokines was higher in cases of dysplasia, although only CD8 and NF-κB showed statistically significant differences. This result suggests that the higher expression of CD8 in the inflammatory infiltrate in dysplasia exercises a protective function, although the maintenance of the stimulus and the differences in expression of other cytokines, such as TNF-α and NF-κB, may favor transformation followed by invasion. While the cases of dysplasia exhibited a lesser quantity of this infiltrate, such cases demonstrated a significant correlation between the intensity of the infiltration and expression of TNF-α in both stromal and epithelial cells and NF-κB.

According to Pacifico and Leonardi (5), the maintenance of activated NF-κB during inflammation predisposes the lesion to malignant transformation. This cytokine is involved in the activation of T-cells, thereby contributing to the transcription of IL-2 and the response of many cell types to pro-inflammatory cytokines, such as TNF (4). In the present study, the overexpression of NF-κB and epithelial TNF-α was positively correlated to the intensity of the inflammatory infiltrate in dysplasia. However, these results are in disagreement with those described by Lind *et al*(20), who noted that the signaling mediated by the TNF-α receptor (TNFR1) in keratinocytes is necessary for the development of skin cancer induced by the inhibition of NF-κB. It was postulated, therefore, that the increased expression of NF-κB in dysplasia and subsequent decrease in carcinoma could be a consequence, rather than the cause, of transformation.

According to Sabel *et al*(6), tumor control depends as much on the magnitude of the initial immune response as the capacity to sustain this response for a prolonged period. In reference to the cases of dysplasia, the present study corroborates this. This strengthens the notion of flexibility regarding the role of inflammation in oral carcinogenesis. This is contrary to the protective role of inflammation used in systems for the histological grading of malignancy proposed by Jakobsson *et al*(21) and Bryne *et al*(22), since the >5% expression of CD8 in 16 (80%) of the 20 cases of OED occurred in only 12 (30%) of the 40 cases of OSCC, with a significant correlation with the intensity of the inflammatory infiltrate.

The inverse correlation between the intensity of the inflammatory infiltrate and epithelial TNF-α in the cases of carcinoma may be responsible for the reduction in NF-κB in epithelial cells following transformation, thereby suggesting a role in this process. On the other hand, the positive correlation between stromal TNF-α and inflammatory infiltrate, which was more intense in the cases of OSCC, suggests a participation in tumor progression. These findings are in disagreement with the possibility of an activated form of NF-κB being induced by various inflammatory stimuli and regulating the gene products (5,23), as this occurred in the cases of dysplasia and was therefore involved in cell transformation. However, these findings are in agreement with the fact that this occurrence is an important link between cancer and inflammation, which may be initiated by the overregulation of TNF-α (24).

In addition, the correlation of TNF-α to the intensity of the inflammatory infiltrate in dysplasia and carcinoma, even while exercising different roles, is in agreement with the findings described by Aggarwal *et al*(25), who suggested that, as one of the major chemical mediators of inflammation, TNF-α is involved in diverse steps of tumorigenesis, including the transformation process.

In normal tissue, TGF-β regulates cell growth and differentiation and functions as a tumor suppressor during the onset of carcinogenesis. In the present study, TGF-β was expressed in a moderate to intense degree in 80% of the cases of OED and in only 60% of the cases of OSCC. However, between the degrees of OED, there was a 50% reduction among the intense cases compared with the mild and moderate cases, all of which had >5% labeling. These findings are in agreement with those described by Glick *et al*(12), who demonstrated the occurrence of carcinomatous transformation in keratinocytes following the autocrine reduction in TGF-β.

According to Donalisio *et al*(13), transformed cells are either partially or totally resistant to the inhibitory effect of TGF-β, which may be related to the degree of cell differentiation. According to Gorelik and Flavell (26), however, in order to favor the antitumor CD8 response, TGF-β must be blocked before the tumor cells partially or totally inhibit this response. It may therefore be considered that, depending on the risk factor, the amount of TGF-β produced after transformation may partially or totally inhibit this antitumor response, leading to the development of a less or more aggressive OSCC, respectively.

In the inflammatory infiltrate, stromal TGF-β was expressed in a moderate to intense degree in 80% of the cases of OED and 82.5% of the cases of OSCC and appears to participate in this process by inhibiting the protective function of CD8, which is in agreement with the findings described by Gorelik and Flavell (26). Thus, TGF-β becomes oncogenic, promoting tumor progression and invasion (14,15).

Sato *et al*(10) report having found a favorable prognosis in cases of ovarian cancer in which the CD8/Treg cell ratio was high. Based on the fact that Treg cells maintained a regular pattern in the cases of dysplasia and carcinoma, with CD8 being more expressed in cases of dysplasia, the results agree with the findings of this previous study and also suggest that a reduction in this ratio favors malignant transformation.

The general context of the present study is in agreement with Weitzman and Gordon (27), who associated a number of chronic inflammatory processes with the development of cancer, as well as Balkwill and Mantovani (28), who suggested the participation of cytokines and inflammatory cells in tumor development and progression. Moreover, the findings are in agreement with Abbas *et al*(19), who report that transformed cells have the ability to evade or overcome the defense mechanisms of the host.

For prognostic evaluation it is necessary to associate a system for the histological grading of malignancy with immunohistochemical analysis to assess both the risk of malignant transformation of dysplasia and tumor aggressiveness. The results of the present study suggest that CD8, TGF-β and TNF-α should be included in this analysis. Further studies with a larger sample size should be carried out in order to confirm the participation of these and other inflammatory cytokines in carcinogenesis and the possibility of their use in antitumor therapy.

In summary, it was concluded that the antitumor reaction exerted by CD8 T-lymphocytes occurs with greater frequency in cases of OED, suggesting a slow down of the transformation and invasion process when associated with a high rate of CD8. The presence of inflammatory infiltrate in cases of OED favors the transformation and invasion process when stromal TNF-α and NF-κB are overexpressed, as NF-κB activated by TNF-α during inflammation predisposes the lesion to transformation, functioning as a link between inflammation and cancer. Although intense inflammatory infiltrate has a positive relation with CD8 in cases of OSCC, the antitumor defense function is minimal, as only ∼30% of cases of carcinoma exhibit >5% labeling. TNF-α, which has a positive correlation in the stroma and a negative correlation in the epithelium when associated to the intensity of the inflammatory infiltrate in cases of OSCC, appears to favor tumor progression following transformation. Transformed keratinocytes either partially or totally lose control of the growth exercised by TGF-β, which becomes oncogenic.

## Figures and Tables

**Table I:** Specifications of primary antibodies.

Antibody	Clone time	Dilution/Incubation	Antigen recovery	Method
NF-κB^a^	NF-κB p65 (A)	1:100/3 h	Citrate	Envision-HRP^b^
TNF-α^a^	TNF-α (2C8)	1:100/Overnight	Pepsin	Envision-HRP^b^
TGF-β^a^	TGF-β1 (V)	1:500/Overnight	Pepsin	Envision-HRP^b^
FOXP3^a^	FOXP3 (H190)	1:100/Overnight	Tris-EDTA, pH 9.0	Envision-HRP^b^
CD8^b^	C8/144B	1:200/60 min	Tris-EDTA, pH 9.0	SABC^b^

a Santa Cruz Biotechnology, Inc. (Santa Cruz, CA, USA);

b Dako (Carpinteria, CA, USA). NF-κB, nuclear factor-κ-light chain enhancer of activated B-cells; TNF-α, tumor necrosis factor-α; TGF-β, transforming growth factor-β; FOXP3, forkhead box P3; CD8, cluster of differentiation 8; HRP, horseradish peroxidase; SABC, Streptavidin-biotin-peroxidase complex.

**Table II:** Correlation between inflammatory infiltrate intensity and expression of cluster of differentiation 8 (CD8), forkhead box P3 (FOXP3), transforming growth factor-β (TGF-β), tumor necrosis factor-α (TNF-α) and nuclear factor-κ-light chain enhancer of activated B-cells (NF-κB).

	Inflammatory infiltrate intensity											

	Dysplasia (n=20)						Carcinoma (n=40)					
	
Expression (%)	Mild n (%)	Mod. n (%)	Int. n (%)	Total	r	P-value	Mild n (%)	Mod. n (%)	Int. n (%)	Total	r	P-value
CD8												
<5	2 (40)	1 (10)	1 (20)	4 (20)			11 (100)	6 (60)	10 (50)	27 (67.5)		
5–50	2 (40)	2 (20)	0 (0)	4 (20)	0.391	NS	0 (0)	1 (10)	2 (10)	3 (7.5)	0.365	<0.05^a^
>50	1 (20)	7 (70)	4 (80)	12 (60)			0 (0)	2 (20)	8 (40)	10 (25)		
FOXP3												
<5	3 (60)	6 (60)	2 (40)	11 (55)			7 (63.6)	6 (60)	11 (57.9)	24 (60)		
5–50	1 (20)	2 (20)	2 (40)	5 (25)	0.109	NS	2 (18.2)	3 (30)	6 (31.6)	11 (27.5)	0.018	NS
>50	1 (20)	2 (20)	1 (20)	4 (20)			2 (18.2)	1 (10)	2 (10.5)	5 (12.5)		
TGF-β (epithelial cells)												
<5	1 (20)	3 (30)	0 (0)	4 (20)			4 (36.4)	3 (30)	9 (47.4)	16 (40)		
5–50	1 (20)	4 (40)	3 (60)	8 (40)	0.026	NS	3 (27.2)	5 (50)	6 (31.6)	14 (35)	−0.142	NS
>50	3 (60)	3 (40)	2 (40)	8 (40)			4 (36.4)	2 (20)	4 (21)	10 (25)		
TGF-β (stromal cells)												
<5	0 (0)	4 (40)	0 (0)	4 (20)			3 (27.2)	2 (20)	2 (10.5)	7 (17.5)		
5–50	2 (40)	1 (10)	2 (40)	5 (25)	0.000	NS	4 (36.4)	3 (30)	8 (42.1)	15 (37.5)	0.189	NS
>50	3 (60)	5 (50)	3 (60)	11 (55)			4 (36.4)	5 (50)	9 (47.4)	18 (45)		
TNF-α (epithelial cells)												
<5	3 (60)	2 (20)	1 (20)	6 (30)			3 (27.3)	1 (10)	10 (52.6)	14 (35)		
5–50	2 (40)	4 (40)	1 (20)	7 (35)	0.442	<0.05^a^	6 (54.5)	6 (60)	6 (31.6)	18 (45)	−0.337	<0.05^a^
>50	0 (0)	4 (40)	3 (60)	7 (35)			2 (18.2)	3 (30)	3 (15.8)	8 (20)		
TNF-α (stromal cells)												
<5	2 (40)	1 (10)	0 (0)	3 (15)			9 (81.8)	3 (30)	9 (47.4)	21 (52.5)		
5–50	3 (60)	6 (60)	1 (20)	10 (50)	0.632	<0.05^a^	1 (9.1)	5 (50)	6 (31.6)	12 (30)	0.383	<0.05^a^
>50	0 (0)	3 (30)	4 (80)	7 (35)			1 (9.1)	2 (20)	4 (21)	7 (17.5)		
NF-κB												
<5	4 (80)	2 (20)	0 (0)	6 (30)			6 (54.5)	6 (60)	8 (42.1)	20 (50)		
5–50	0 (0)	0 (0)	0 (0)	0 (0)	0.617	<0.05^a^	3 (27.3)	2 (20)	10 (52.6)	15 (37.5)	0.046	NS
>50	1 (20)	8 (80)	5 (100)	14 (70)			2 (18.2)	2 (20)	1 (5.3)	5 (12.5)		

Spearman rank correlation coefficient.

a P<0.05, statistically significant; NS, not significant. Mod., moderate; Int., intense.
